# Mapping the Edges of Mass Spectral Prediction: Evaluation of Machine Learning EIMS Prediction for Xeno Amino Acids

**DOI:** 10.1021/acs.analchem.5c00286

**Published:** 2025-05-07

**Authors:** Sean M. Brown, Evan Allgair, Robin Kryštůfek

**Affiliations:** † Department of Biological Sciences, 14701 University of Maryland, Baltimore County, Baltimore, Maryland 21250, United States; ‡ 89220 Institute of Organic Chemistry and Biochemistry of the Czech Academy of Sciences, 160 00 Praha 6-Dejvice, Czechia

## Abstract

Mass spectrometry is one of the most effective analytical methods for unknown compound identification. By comparing observed *m*/*z* spectra with a database of experimentally determined spectra, this process identifies compound(s) in any given sample. Unknown sample identification is thus limited to whatever has been experimentally determined. To address the reliance on experimentally determined signatures, multiple state-of-the-art MS spectra prediction algorithms have been developed within the past half decade. Here we evaluate the accuracy of the NEIMS spectral prediction algorithm. We focus our analyses on monosubstituted α-amino acids given their significance as important targets for astrobiology, synthetic biology, and diverse biomedical applications. Our general intent is to inform those using generated spectra for detection of unknown biomolecules. We find predicted spectra are inaccurate for amino acids beyond the algorithms training data. Interestingly, these inaccuracies are not explained by physicochemical differences or the derivatization state of the amino acids measured. We thus highlight the need to improve both current machine learning based approaches and further optimization of *ab initio* spectral prediction algorithms so as to expand databases for structures beyond what is currently experimentally possible, even including theoretical molecules.

## Graphical abstract

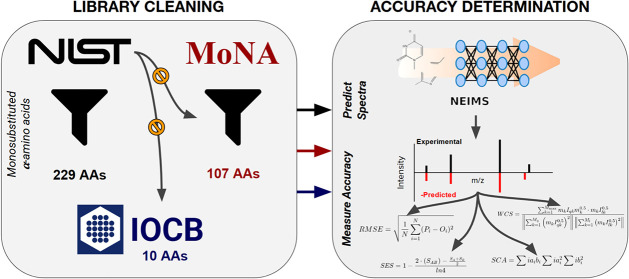

## Introduction

Mass spectrometry (MS) is widely accepted as one of the most effective analytical methods for identifying unknown compounds. Widespread use of MS ranges from multiple research frontiers (*e.g.*, from toxicology^1^ to astrobiology^2^) to education.^3^ This ubiquity can be attributed to the high sensitivity and specificity of electron–ionization (EI) mass spectrometry, especially when paired with gas chromatography (GC).^4–6^ Unknown compounds can be identified with MS by measuring the mass-to-charge ratio (*m*/*z*) of ions in a sample. This data is typically represented by an intensity plot of *m*/*z* values in the form of a spectrum. For unknown compound identification, the observed spectra are compared with a library of known spectra^7–9^ so as to identify the compound(s) in a sample.

Amino acids are a notable example of small biomolecules often identified through MS. Genetically encoded amino acids comprise a central α-carbon with variable side-chains (R-groups) in between amino (−NH_2_) and carboxyl (−COOH) termini. Amino acids are also the primary constituents of the chemical network biologists know as metabolism, in the form of proteins. Life on Earth, for the last 3.5 billion years, has functioned as a network of genetically encoded proteins: each protein comprises a polymerized sequence of amino acids. Despite the availability to life’s origins and early evolution of many alternative amino acids,^10–12^ extant life assembles genetically encoded proteins with a standard alphabet of 20 l-α-amino acids. Given that amino acids are foundational to the “*N* = 1” example of life as we know it, they are a clear target when looking for life elsewhere in the cosmos.^11^

Gas chromatography mass spectrometry (GCMS) is central to *in situ* extraterrestrial life detection for variety of reasons:^13^ compatibility with solid, liquid, and gas samples;^14^ detecting enantiomeric excess;^15^ high-sensitivity detection of trace compounds within a sample;^16^ and successful miniaturization for spaceflight.^17,18^ Examples of this widespread usage include (i) the ESA/Roscosmos ExoMars’s GCMS within the Mars Organic Molecule Analyzer (MOMA)^14^ (ii) the Europa Clipper Mission which is equipped with the MAss Spectrometer for Planetary EXploration/Europa (MASPEX)^19^ and (iii) the Dragonfly Mission’s GCMS (DraMS).^20^

A notable limitation of unknown compound identification via MS is, therefore, the reliance on previously established reference data, usually in the form of MS spectral databases. In other words, if a compound’s spectrum does not exist within any known database, then compound identification becomes intimidating. In an attempt to address this limitation, multiple state-of-the-art MS spectra prediction algorithms have been developed within the past half decade.^21^ One approach to predict spectra, seen in NEIMS^22^ or RASSP,^23^ trains a machine learning (ML) algorithm on a library of experimentally determined spectra. NEIMS, the algorithm evaluated here, for instance was trained on EI-MS spectra from roughly 300,000 molecules. After training and validation, these algorithms are reported to quickly predict spectra with varying degrees of accuracy.^21^ This rapidly growing use of machine learning has replaced the traditional approach of building a ruleset, such as seen in CFM-EI 3.0,^24^ from which the fragmentation pattern of a sample can be estimated.

The other major contemporary approach to predict MS spectra uses physics-based (*ab initio*) models. Instead of using machine learning to predict EI-MS spectra, QCxMS^25,26^ for example, applies semiempirical quantum mechanics modeling to predict the fragmentation patterns of a molecule. Unlike ML models, *ab initio* models do not require a training data set, but only at the expense of substantially increased computational time. Algorithms such as QCxMS are therefore best suited for prediction of molecules with fewer than 50 atoms. To take an example, the DFT-D3 runtimes “for a midsized organic molecule (50 atoms) takes...days to weeks”^27^ When shifting from DFT methods to semiempirical quantum mechanical (SQM) methods which QCxMS allows (*e.g.*, GFN2-xTB—the default level used in QCxMS), the computational cost is significantly reduced. For example, a QCxMS simulation for norleucine completes within several hours on a single CPU core and can even be completed quicker with parallelization. In this sense, for at least some of the smaller unmodified amino acids analyzed here, QCxMS calculations at the GFN2-xTB level are computationally feasible and could, in principle, be performed for comparison. However, for the larger, especially derivatized, amino acids the computational requirements remain too high when analyzing hundreds of molecules (see Supporting Information). Therefore, while GFN2-xTB level simulations are possible for small molecules, we conclude that they remain outside the practical scope of this study.

Previous reviews of MS spectra prediction have focused on either a wide range of chemical classes^21^ or have narrowly evaluated the prediction accuracy of a single chemical class such as Purines and Pyrimidines.^28^ However, no literature explicitly targets the accuracy of these algorithms for predicting amino acid MS spectra. Given the significance of amino acids as important and tractable targets for astrobiology,^29^ synthetic biology,^30^ and diverse biomedical applications,^31^ here we therefore evaluate the accuracy of the NEIMS (v.1.0) machine learning spectral prediction algorithm for monosubstituted α-amino acids found both within, and beyond the algorithm’s native training set.

## Results

This study evaluated the accuracy of NEIMS EI-MS spectral prediction for monosubstituted α-amino acids found within three spectral libraries: the National Institute of Standards and Technology’s 2017 mass spectral data set (NIST17), the Mass Bank of North America’s Mass Spectral Database (MoNA) specifically filtered to exclude spectra also found within the NIST17 database (MoNA_f_), and a hand curated set comprising GCMS spectra for amino acids outside NIST17 (hereinafter IOCB). NIST 17 comprises “a fully evaluated collection of electron ionization (EI)...mass spectra”.^32^

For each predicted amino acid spectrum within each MS library, four measurements of accuracy were calculated (also see Figure SI.1 and eqs 1–2): padded spectral RMSE, spectral contrast angle (SCA), weighted cosine similarity (WCS), and spectral entropy similarity (SEN). Further measurements then sought to establish the cause of discrepancies between predicted and experimental spectra in terms of (i) physicochemical differences (molecular weight and hydrophobicity) and (ii) *N*-(*tert*-butyldimethylsilyl)-*N*-methyltrifluoroacetamide (MTBSTFA) derivatization. Results show that neither inherent physicochemical properties nor chemical modification account for the inaccuracy of spectral prediction by any of the measures tested in any of the three libraries as described below.

Figure 1 shows that the NEIMS algorithm, across all four accuracy metrics and both libraries comprising molecules outside of the algorithm’s training set (MoNA_f_ and IOCB), fails to consistently predict reliable EIMS spectra of amino acids by our measures. Specifically, amino acids within the NEIMS training set (NIST17) display the highest accuracy with mean RMSE, SCA, WCS, and SEN values of 9.64, 37.3°, 0.84, and 0.80 respectively. The accuracy of predicted amino acid spectra within the IOCB library followed those within NIST17 with mean RMSE, SCA, WCS, and SEN values of 13.0, 64.7°, 0.63, and 0.58 respectively. The lowest accuracy recorded was therefore the predicted amino acid spectra within MoNA_f_, with mean RMSE, SCA, WCS, and SEN values of 12.7, 82.1°, 0.20, and 0.20 respectively.

**Figure 1:**
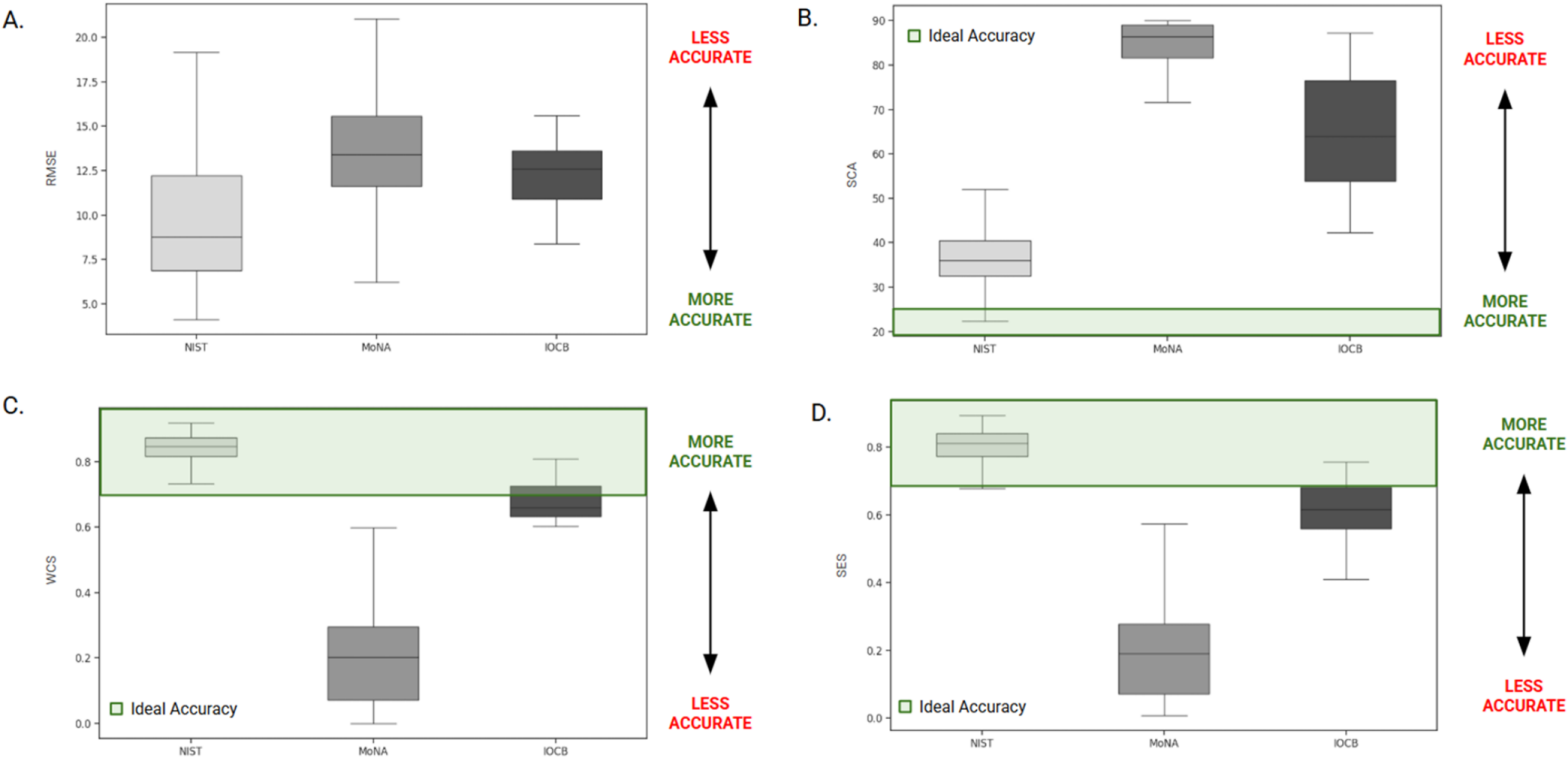
NEIMS predicted EI-MS spectra accuracy. Accuracy measurements span three spectral libraries of amino acids (i) NIST-MS 2017 (ii) MoNA_f_ (iii) a hand curated set of amino acids (IOCB). Accuracy for predicted spectra is measured in terms of (A) spectral root-mean-square error (RMSE), (B) spectral contrast angle (SCA), (C) weighted cosine similarity (WCS), and (D) spectral entropy similarity (SEN). For SCA, angles below ∼26° (shown in green) are typically adequate for library search algorithms.^33^ For WCS, the metric used as the training metric within the NEIMS algorithm, scores greater than 0.7 (shown in green) are generally classified as “similar”.^34^ For SEN, scores greater than 0.75 are considered ideal as “false discovery rates of less than 10%” with scores of 0.75 or greater.^35^

A deeper understanding of these results comes from measuring the impact on accuracy of differences in physicochemical properties and derivatization (Table SI.1). Figure 2 shows no clear effect of molecular weight or hydrophobicity on prediction accuracy for any metric or library. Low correlations between hydrophobicity and all accuracy metrics, calculated as coefficients of determination (*R* ^2^), range from 0.01 to 0.13 across all libraries (Table SI.2). Similarly low correlations between molecular weight and accuracy typically range from 0.01 to 0.15 across each library and accuracy metric. The one exception of IOCB/RMSE (*R* ^2^ = 0.38) seems likely to reflect the small sample size of the IOCB library in that it shows a counterintuitive negative correlation: larger, more complex molecules are easier to predict.

**Figure 2:**
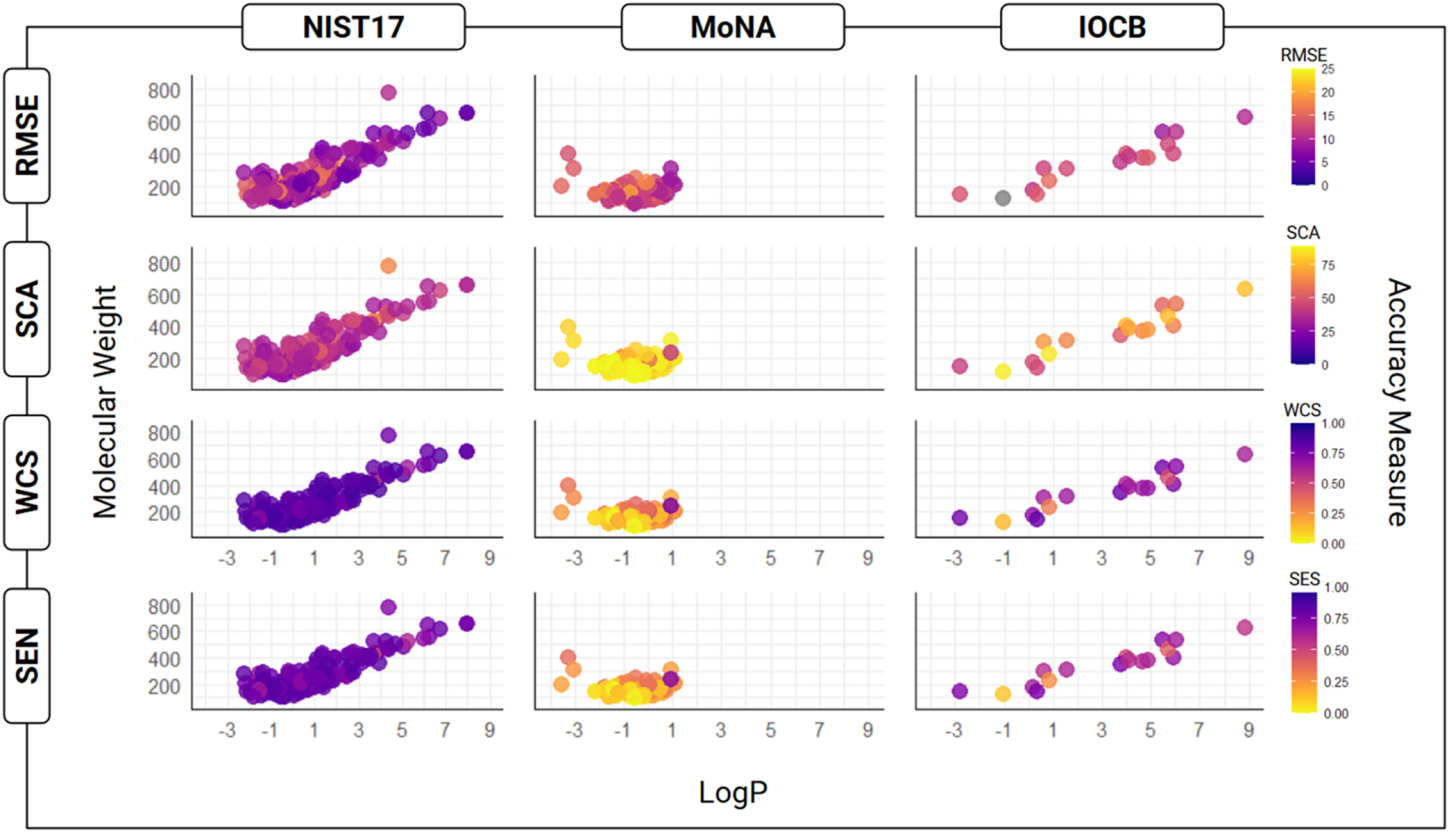
Accuracy across chemical space. Prediction performance is evaluated across three libraries (Columns: NIST17, MoNA, IOCB) with four accuracy metrics (Rows: RMSE, WCS, SCA, SEN). These are plotted as a function of chemical space (Molecular Weight, JChem log *P*). Accuracy in each plot is color coded from purple (most accurate) to yellow (least accurate) for each respective metric. The physicochemical properties we consider reveal no clear clustering patterns.

A final measurement compared the four accuracy metrics against underivatized and MTBSTFA derivatized amino acids in each library. Figure 3 demonstrates that, surprisingly, derivatized amino acids do not differ significantly from underivatized amino acids in terms of accuracy metrics across libraries. Specifically, we observe *t* test *p*-values ranging from 0.19–0.93 (Table SI.3) with the notable exception of NIST/RMSE with a *p*-value of 9.3 × 10^–9^. In other words, the predicted spectra for underivatized amino acids within the NIST library produced a significantly broader range prediction accuracy when measured by RMSE than all other distributions considered. It completely eludes us why free amino acids in this sample, measured in this way, should be so different from free amino acids in other samples given that the IOCB library covers much the same physicochemical range as the NIST library.

**Figure 3:**
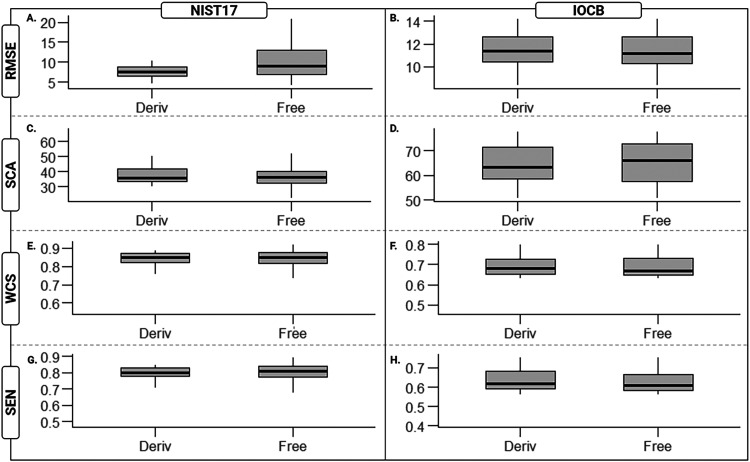
Accuracy measured by RMSE, SCA, WCS, SEN for underivatized and MTBSTFA derivatized amino acids. Accuracy metrics are compared across MTBSTFA derivatized (Deriv) and underivatized (Free) amino acids for the NIST17 and IOCB libraries. (A) Amino acid spectra within the NIST17 library, when accuracy is measured via RMSE, display a significant difference in accuracy (*t* test *p*-value <0.001) between MTBSTFA derivatized (Deriv) and underivatized (Free) amino acid spectra. All other comparisons (B–H) are found to be not significantly different (*t* test *p*-value >0.05). The MoNA database was omitted from this analysis as there were no unique MTBSTFA monosubstituted α-amino acid spectra within MoNA.

## Discussion

We evaluated the accuracy of the NEIMS (v.1.0) machine learning spectral prediction algorithm for monosubstituted α-amino acids found both within, and beyond the algorithm’s training set. We did so by measuring the accuracy of RMSE, SCA, WCS, and SEN. Our intent is to inform researchers who rely on predicted spectra for detection of unknown biomolecules, specifically amino acids. Results indicate that current machine learning algorithms are probably insufficient to predict spectra accurately.

While NEIMS performs well for the molecules and measures for which it was trained, accuracy declines for molecules (amino acids) outside of the training set and when accuracy is measured in other ways (SCA/RMSE). Despite training on over 300,000 molecules within the NIST mass spectral database. The pattern was consistent across all four accuracy metrics and all three libraries tested, albeit to varying degrees. This finding is perhaps intuitive given the small proportion of the NIST-MS amino acid spectra (∼0.01%), but here we quantify to what degree this problem manifests. More surprisingly the data also show that neither derivatization nor physicochemistry (molecular weight and hydrophobicity), correlate with accuracy. While this might follow from the small fraction of amino acids within the NEIMS training set, it gives no clear insight into why NEIMS struggles to predict MS spectra for these amino acids reliably. In terms of derivatization, the enigma is that the NEIMS algorithm is just as accurate for “free” amino acids as for their derivatized counterparts. Because MTBSTFA derivatization is designed to standardize the ionization and fragmentation pattern of amino acids: one could anticipate it would create a more consistent framework for predictive modeling. On the other hand, by replacing active hydrogens in hydroxyl, amino, and thiol groups with *tert*-butyldimethylsilyl (TBDMS), derivatization adds atoms and complexity which could produce the opposite effect. In fact we see neither: derivatized and free amino acids are indistinguishable in terms of prediction accuracy.

Similarly, one could plausibly anticipate that larger, more complex, amino acids would present a greater challenge to machine learning as more noisy spectra provide a challenging space for ML algorithms to distinguish patterns. We do not observe this. Rather we see a lack of correlation between size and prediction accuracy, and even (for one measurement and library) the opposite. As for polarity/hydrophobicity, intuition is less clear. Again, however, the empirical data show no discernible pattern between this physicochemical property and prediction accuracy.

## Conclusions

Mass spectrometry for compound identification relies on established databases. Once MS spectral prediction algorithms are validated, there is a critical need for comprehensive libraries of predicted spectra for unknown and theoretical molecules (amino acids). Wherever reliable theoretical databases of predicted mass spectra can be formed, they will allow for greater expansion of potential search space for an unknown molecule in a sample. Such tools would have broad uses from informing NASA mission data (*e.g.*, Mars Sample Return^36^) to expanding public health surveillance.^37^ For instance, the Robert Koch Institute has designed a MALDI-TOF MS database specifically to identify pathogenic bacteria (ibid) and the National Institute of Standards and Technology (NIST) has begun modeling fragmentation energetics to enable unknown compound identification through theoretical spectra.^38^ Beyond amino acids, predictions that allow us to extend to other classes of biomolecules (*e.g.*, lipids, sugars, or nucleic acid derivatives) would further advance many disciplines.

Our findings highlight noteworthy limitations in current machine-learning-based MS spectral prediction algorithms. This inaccuracy underscores motivation for future research as follows. Given that NEIMS is less accurate for amino acids beyond those used for model training, there is a need to understand better the cause and solution of current limitations. One simple solution for amino acids could be to retrain NEIMS with a larger or targeted training set (*e.g.*, amino acid spectra curated within these analyses). While this could work for specific applications with targeted analytes of interest, this approach will always be incremental given the current broad applicability of NEIMS. With this in mind, our findings further display the need to optimize existing *ab initio* prediction algorithms (*e.g.*, QCxMS^25^) for larger molecules. If GCMS spectra prediction could readily be trusted for any molecule, even for those outside of a ML algorithm’s training set, then reliable databases could be curated for any structures currently missing spectra, even theoretical molecules (*e.g.*, AACL amino acid library^39^).

## Experimental Methods

### Mass Spectral Prediction Algorithms

These analyses used NEIMS (version 1.0)^22^ to calculate the mass spectral profiles for monosubstituted α-amino acids. Instructions on installation and setup of NEIMS can be found at https://github.com/brain-research/deep-molecular-massspec. We used the NEIMS pretrained machine learning model weights trained on nearly 300,000 EI-MS spectra within the NIST MS 2017 database. Other prediction algorithms do exist in the literature for simulating EI-MS spectra, however we focus our efforts on NEIMS for numerous reasons. CFM-EI^40^ has depreciated support for electron ionization prediction in the latest version (CFM-ID 4.0)^24^ and our efforts failed to compile previous versions published by developers despite troubleshooting efforts. RASSP^23^ was not included in this study due to repeated compilation failures across tested environments, specifically with errors commonly encountered by other developers (*e.g.*, see https://github.com/thejonaslab/rassp-public) pertaining to the RASSP environment file. Despite extensive efforts to resolve these issues, including manually installing dependencies, RASSP could not be successfully implemented for evaluation. All *m*/*z* values of the data sets are rounded to integer masses, given that NEIMS is only able to predict integer masses.

QCxMS^25^ was jettisoned from this analysis as well, simply due to runtimes required for larger, especially MTBSTFA derivatized, amino acids (see Figure SI.2). For example, we observed a runtime for “free” norvaline (19 atoms) over 50 h on the tested systems (Linux Ubuntu 22.04.3 LTS, AMD Ryzen 5 3600 12 Core 4.2 GHz CPU). All QCxMS simulations were performed using GFN2-xTB with *n*_traj_ = 25 × N_atoms, *t*_step_ = 0.5 fs, and *t*_max_ = 5.0 ps, electron impact energy = 70 eV, and initial temperature equaling 500 K. The system was allowed to equilibrate for 2 ps prior to impact.

### Libraries of GC-EI-MS Spectra

We investigated three libraries for this study (i) the spectra found within the NEIMS training set (NIST17) (ii) an open source repository filtered to remove duplicate spectra from NIST17—MoNA_f_ and (iii) the IOCB hand-curated data set. As previously mentioned, the NEIMS neural network was trained on ∼300,000 EI-MS spectra present in the NIST 2017 database. Of these ∼300,000 spectra, monosubstituted α-amino acids comprise only ∼0.01% of the training data. Therefore, for the purposes of this study, we filtered the molecules in NIST to only contain Type Ia^39^ monosubstituted α-amino acids (*N* = 229; see Supporting Information).

The second data set investigated here was the MassBank of North America (MoNA).^7^ MoNA is an open-source “auto-curating repository” for mass spectral records. We then filtered the GC-MS subset of 18,915 spectra to only contain Type Ia monosubstituted α-amino acids measured by GC-EI TOF not found in the NEIMS training set (MoNA_f_).

### Spectral Prediction Accuracy Measures

We used four separate measures of spectral similarity to evaluate the accuracy of NEIMS (i) Root Squared Mean Error (ii) Spectral Contrast Angle (iii) Weighted Cosine Similarity, and (iv) Spectral Entropy Similarity. All accuracy metrics below were calculated in Python^41^ (version 3.12.2) along with libraries rdkit-pypi^42^ (version 2022.9.5), pandas^43^ (version 2.2.2), numpy^44^ (version 1.26.4).

Root Squared Mean Error

1 $$\mathrm{RSME}=\sqrt{\frac{1}{N}\sum_{i=1}^{N}{\left(P_{i}-O_{i}\right)}^{2}}$$

RMSE is a common metric especially when determining the accuracy of machine learning models.^45^ Here, we used a RMSE calculation as one metric of aligned, or “zero-padded”, spectral similarity (eq 1 and Figure SI.1). The RMSE calculation function begins with two numerical lists of the experimental and predicted spectrum. These two lists are then “zero-padded” from *m*/*z* = 0 to maximum *m*/*z* of either spectrum. The change of intensity for each *m*/*z* integer, “ΔIntensity”, is then incorporated into the function above where ΔIntensity equals *P*_*i*_ – *O*_*i*_.

Spectral Contrast Angle

$$\mathrm{SCA}=\cos\theta =\Sigma a_{i}b_{i}/\sqrt{\Sigma {a_{i}}^{2}\Sigma {b_{i}}^{2}}$$ 2

Spectral contrast angle (eq 2) determines spectral similarity by representing two spectra in an N-dimensional space by two vectors. Where “*a_i_* and *b_i_* are the relative intensities of product-ion peaks at *m*/*z* value *i* for isomers A and B”.^46^ Thus, the measured angle between these two vectors quantifies spectral similarity from 0 to 90° (where 0° indicates indistinguishable spectra). SCA, given the focus on the angle between, rather than the magnitude of vectors, is less sensitive to differences in peak intensity. In other words, SCA weighs each peak equally when calculating the similarity between two spectra. When using SCA to measure accuracy, angles above ∼26° often are not adequate for library search algorithms.^33^

We furthermore calculated the weighted cosine similarity (WCS) score which is a common metric used by mass spectrometry software^47^ and is precisely how the creators of NEIMS, Wei et al., measured spectral similarity when training the NEIMS machine learning algorithm.^22^ WCS measures the cosine of the angle between two vectors in a multidimensional space where each vector represents a spectra and the dimensions correspond to intensities at each *m*/*z* value. In terms of accuracy, WCS scores greater than 0.7 are generally classified as “similar”.^34^

The final accuracy metric calculated is spectral entropy similarity^35,48^ (SEN) which is a novel mass spectra similarity metric. SEN specifically measures the informational similarity of two spectra rooted in information theory’s Shannon Entropy.^49^ Spectral entropy similarity has been shown to have a greater sensitivity than dot-product methods for spectral similarity matching outperforming forty-two similarity metrics.^35^ Furthermore, this metric has been developed so as to quickly compare spectra against “large spectral libraries with little memory overhead for any mass spectrometry laboratory.”^48^ With SEC, scores greater than 0.75 are generally classified as “similar” as “false discovery rates of less than 10%” with scores of 0.75 (ibid).

### Preparation and GC EI-MS Analysis of IOCB Data Set

The last data set, IOCB, is a hand-curated data set of experimentally measured GCMS TOF spectra for both free and MTBSTFA derivatized amino acids not found in NIST. The derivatization reagent was a mixture of MTBSTFA (≥98.0% Merck 77626) and DMF (≥99.9% VWR 83634.320). Amino acids were prepared by deprotecting commercially available Fmoc-protected amino acid blocks obtained from Iris Biotech (FAA1210, FAA1368, FAA6800, FAA1195, FAA7030, FAA5600, FAA1920, FAA3270, FAA2690) and Santa Cruz Biotechnology (sc-285835). Protected amino acids were treated with a mixture of TFA/TIS/water (95:2.5:2.5) for 2 h for preparations without side chain protection. After this, the mixture was removed under reduced pressure, and the residue was treated with 20% piperidine in DMF for 30 min. The crude product was purified by RP HPLC on a Luna 5 μm C18(2) 100 Å, 250 mm × 21.2 mm column (Phenomenex 00G-4252-P0-AX) using a 0–45% gradient (A: 10 mM TEAA buffer, B: ACN) with an initial 15 min 0% B hold to remove residual DMF. Target fractions were collected, lyophilized, and optionally treated with 60:20 MTBSTFA/DMF (200 μL per 50 μg of analyzed compound) at 80 °C for 1 h.^50^ The GC EI-MS data were measured on a Agilent 7250 Accurate-Mass Quadrupole Time-of-Flight GC/MS System with a 15 m J&W HP-5 ms Ultra Inert GC Column (Agilent 19091S-431UI). The ionization energy was set to 70 eV to comply with the specifications at which NIST spectra are measured,^51^ and was achieved with an electron extractor voltage of 15.0 V and a repeller voltage of 21.0 V. The source temperature was maintained at 230 °C with an emission current of 0.5 μA. The instrument operated in high-resolution combined mode (EDR-TLPP), with a mass acquisition range from *m*/*z* 50 to 1200 at a rate of 1 Hz (1000 ms/spectrum). The mass spectrometer was tuned using perfluorotributylamine (PFTBA) reference masses with subppm mass accuracy. Spectrum at the dominant TIC peak of the measured chromatogram was used for subsequent analysis.

Figures throughout this manuscript were generated using R (version 4.2.2), Python^41^ (version 3.10.12), along with the ggplot2 ^52^ (version 5.3.1), matplotlib^53^ (version 3.8.0), and seaborn^54^ (version 0.13.2) packages.

## Supplementary Material



## Notes

Additional experimental details, materials, and methods, and data including: library characterization, amino acid filtering, and the libraries (PDF)
