# Supplementary material for: Mapping the Edges of Mass Spectral Prediction: Evaluation of Machine Learning EIMS Prediction for Xeno Amino Acids
Source: Anal Chem. 2025 May 7;97(19):10282–8. doi: 10.1021/acs.analchem.5c00286 (PMC12096351; doi:10.1021/acs.analchem.5c00286)
Supplement: Supplementary file 1 [file ac5c00286_si_001.pdf]

Supplementary Information

Mapping the Edges of Mass Spectral Prediction: Evaluation of Machine Learning EIMS Prediction for Xeno Amino Acids

Sean M. Brown[1]*, Evan Allgair[1], & Robin Kryštůfek[2]

[1]University of Maryland, Baltimore County: Department of Biological Sciences, Baltimore, Maryland, 21250

[2]Institute of Organic Chemistry and Biochemistry of the Czech Academy of Sciences, 160 00 Praha 6-Dejvice, Czechia

*Author to whom correspondence should be addressed.

TABLE OF CONTENTS

SI.1 Library Characterization 3
SI.2 QCxMS Benchmarking 4
SI.3 Amino Acid Filtering 5
SI.4 NIST17, MoNAf, & IOCB Libraries 5

SI.1 Library Characterization

Table SI.1: Physicochemical property characterization of libraries

Library	N Mols	μ Mol Wt	μ LogP	μ Aromatic Proportion	μ Rotatable Bonds
**NIST**	229	245	1.22	0.24	5
**$MoNA_f$**	107	165	-0.41	0.11	4
**IOCB**	10	279	2.17	0.06	5

Table SI.2: Correlation Values (R^2) Between Physicochemical Properties and Accuracy

Library		MW	LogP
	RMSE	0.05	0.13
	SCA	0.04	0.01
	Cosine Similarity.	0.02	0.03
**NIST**	*Spectral Entropy*	0.03	0.04
	RMSE	0.01	0.12
	SCA	0.10	0.13
	Cosine Similarity	0.13	0.09
**$MoNA_f$**	*Spectral Entropy*	0.15	0.10
	RMSE	0.38	0.28
	SCA	0.04	0.05
	Cosine Similarity	0.06	0.04
**IOCB**	*Spectral Entropy*	0.04	0.02

Table SI.3: T-Test P-Values Between Free and MTBSTFA Derivatized Amino Acid Spectra

Library		*P*
	RMSE	9.3×10^{-9}
**NIST**	*SCA*	0.35

	Cosine Similarity.	0.35
	Spectral Entropy	0.19
	RMSE	0.87
	SCA	0.93
	Cosine Similarity	0.90
**IOCB**	*Spectral Entropy*	0.88

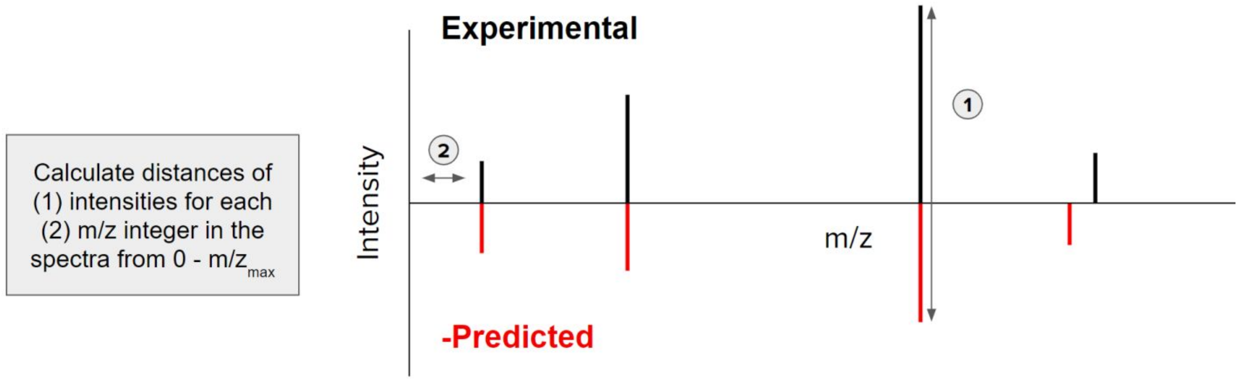

Figure SI.1. Calculation of RMSE for Spectra Comparison. RMSE is calculated from the intensity differences between the aligned (zero-padded) predicted and experimental spectra across the m/z range. Zero padding simply adds an intensity value of 0 at each integer between measured m/z values.

SI.2 QCxMS Benchmarking

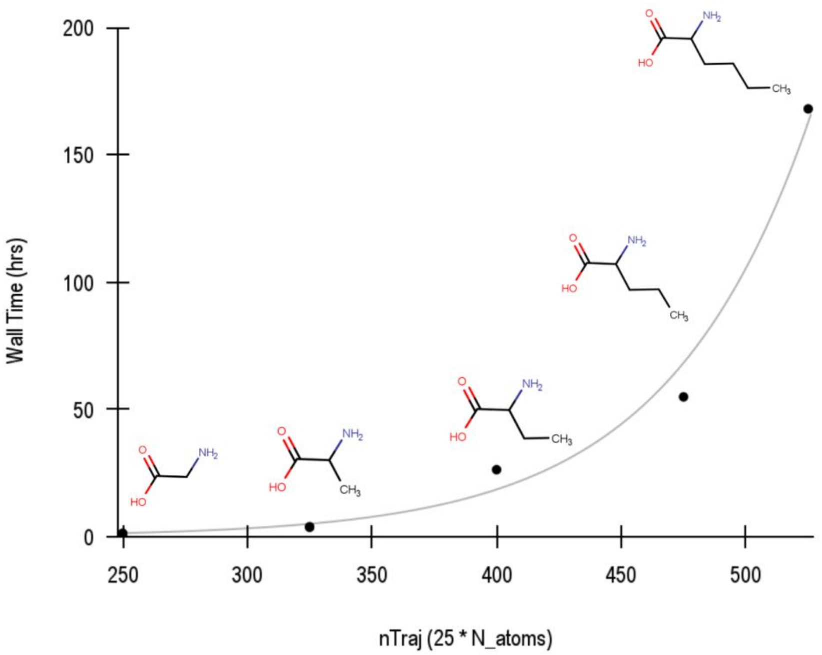

Figure SI.2. Benchmarking QCxMS. To benchmark the feasibility of QCxMS (GFN2-xTB Hamiltonian D4-dispersion) for the purposes of this study, we computed five ‘free’ amino acids (glycine, alanine, α-Aminobutyric acid, norvaline, and norleucine) EIMS spectra within QCxMS. For molecules larger than glycine, computational runtime expectedly increases exponentially as the number of atoms increases (represented as the number of trajectories - nTraj). Larger, especially MTBSTFA amino acids, require immense computational resources and thus QCxMS was jettisoned from the analysis presented here. The correlation coefficient (R2) for the trendline ($y = 0.0177e^{0.0174x}$) displayed equals 0.99.

SI.3 Amino Acid Filtering

For the NEIMS Training Set molecules (NIST17), we first downloaded a SMILES list of source molecules used for training the NEIMS mass spectra prediction machine learning model provided in Wei et al. (2017) supplementary information. From here Python scripts, along with cheminformatics packages such as RDKit, were used to filter this dataset for monosubstituted α-amino acids. These scripts simply matched free and MTBSTFA derivatized amino acids via a series of SMARTS strings representing the amino acid backbone. Once the amino acids were subsetted, EI-MS spectra are then manually matched from the NIST-MS database using the smiles strings of the selected amino acids.

Similarly for the $MoNA_f$ dataset, the entirety of the MoNA Databank was downloaded and filtered by instrument to only include monosubstituted α-amino acids spectra measured by GC-EI TOF. Finally, any spectra from monosubstituted α-amino acids within the MoNA database that were also present in the NEIMS training dataset were removed using similar python scripts to those mentioned above.

SI.4 NIST17, MoNAf, & IOCB Libraries

Table SI.3: Libraries of amino acids comprising the molecule name and SMILES string for each entry.

Library	Molecule Name	SMILES
NIST17	*6-Methyl-DL-tryptophan*	Cc1ccc2c(CC(N)C(=O)O)c[nH]c2c1
NIST17	*N.omega.-Nitro-L-arginine*	N=C(NCCC[C@H](N)C(=O)O)N[N+](=O)[O-]
NIST17	*L-Serine, dihydrogen phosphate*	N[C@@H](COP(=O)(O)O)C(=O)O
NIST17	*5-Fluorotryptophan*	NC(Cc1c[nH]c2ccc(F)cc12)C(=O)O
NIST17	*N(5)-carbobenzyloxy-dl-4-hydroxyornithine*	NC(CC(O)CNC(=O)OCc1ccccc1)C(=O)O
NIST17	*2-Formyl-l-tryptophan*	N[C@@H](Cc1c(C=O)[nH]c2ccccc12)C(=O)O
NIST17	*L-Homoserine, O-propyl-*	CCCOCC[C@H](N)C(=O)O
NIST17	*Benzenepropanoic acid, .alpha.-amino-4-(trifluoromethoxy)-*	NC(Cc1ccc(OC(F)(F)F)cc1)C(=O)O
NIST17	*Dansyl-l-histidine*	CN(C)c1cccc2c(S(=O)(=O)n3cnc(C[C@H](N)C(=O)O)c3)cccc12
NIST17	*S-Benzyl-dl-cysteine*	NC(CSCc1ccccc1)C(=O)O
NIST17	*Histidine, 4-trifluoromethyl-*	NC(Cc1[nH]cnc1C(F)(F)F)C(=O)O
NIST17	*3-(3-Hydroxy-4-methoxyphenyl)-l-alanine*	COc1ccc(C[C@H](N)C(=O)O)cc1O
NIST17	*L-Homoserine, O-ethyl-*	CCOCC[C@H](N)C(=O)O
NIST17	*5-Hexynoic acid, 2-amino-4-(hydroxymethyl)-*	C#CC(CO)CC(N)C(=O)O
NIST17	*d-Threo-O-ethylthreonine*	CCOC(C)C(N)C(=O)O
NIST17	*2-Amino-6-benzamidohexanoic acid*	NC(CCCCNC(=O)c1ccccc1)C(=O)O
NIST17	*N(Epsilon)-methyl-l-lysine*	CNCCCC[C@H](N)C(=O)O
NIST17	*S-Carboxymethyl-L-cysteine*	N[C@@H](CSCC(=O)O)C(=O)O
NIST17	*DL-Methionine sulfone*	CS(=O)(=O)CCC(N)C(=O)O
NIST17	*Phenylalanine, 3-methoxy-*	COc1cccc(CC(N)C(=O)O)c1
NIST17	*D-tert-Leucine*	CC(C)(C)[C@@H](N)C(=O)O
NIST17	*L-Alanine, 3-[(aminocarbonyl)amino]-*	NC(=O)NC[C@H](N)C(=O)O

NIST17	*Erythro-p-amino-.beta.-hydroxy-dl-phenylalanine*	Nc1ccc(C(O)C(N)C(=O)O)cc1
NIST17	*Indole-3-propionic acid, .alpha.-amino-5-chloro-*	NC(Cc1c[nH]c2ccc(Cl)cc12)C(=O)O
NIST17	*D-Ethionone*	CCSCC[C@@H](N)C(=O)O
NIST17	*S-(7-Methyl-1-naphthyl)cysteine*	Cc1ccc2cccc(SCC(N)C(=O)O)c2c1
NIST17	*3-(3-Cyano-4-hydroxyphenyl)-l-alanine*	N#Cc1cc(C[C@H](N)C(=O)O)ccc1O
NIST17	*3-Pyrrolidineacetic acid, .alpha.-amino-5-carboxy-2-oxo-, [3R-[3.alpha.(S*),5.alpha.]]-*	NC(C(=O)O)C1CC(C(=O)O)NC1=O
NIST17	*S-Tetrahydropyran-2-yl-cysteine*	NC(CSC1CCCCO1)C(=O)O
NIST17	*α-Amino-4-chlorobenzeneacetic acid*	NC(C(=O)O)c1ccc(Cl)cc1
NIST17	*Histidine, 2-fluoro-, L-*	NC(Cc1cnc(F)[nH]1)C(=O)O
NIST17	*3-[3,4-Methylenedioxyphenyl]alanine*	NC(Cc1ccc2c(c1)OCO2)C(=O)O
NIST17	*2-Amino-3-(propylthio)propionic acid*	CCCSCC(N)C(=O)O
NIST17	*Amino(bicyclo[6.1.0]non-4-en-9-yl)acetic acid*	NC(C(=O)O)C1C2CC/C=C\CCC21
NIST17	*dl-Lysine*	NCCCCC(N)C(=O)O
NIST17	*Norvaline, 3-hydroxy-*	CCC(O)C(N)C(=O)O
NIST17	*4-Fluorohistidine*	NC(Cc1[nH]cnc1F)C(=O)O
NIST17	*2,3-dihydro-tryptophan*	NC(CC1CNc2ccccc21)C(=O)O
NIST17	*2-Nitrotryptophan*	NC(Cc1c([N+](=O)[O-])[nH]c2ccccc12)C(=O)O
NIST17	*.beta.-[5-Methyl-2-tetrahydrofuranyl]alanine*	CC1CCC(CC(N)C(=O)O)O1
NIST17	*Cysteine, S-methyl-*	CSCC(N)C(=O)O
NIST17	*Glycine, 2-(m-hydroxyphenyl)-, DL-*	NC(C(=O)O)c1cccc(O)c1
NIST17	*Benzenebutanoic acid, .alpha.,2-diamino-.gamma.-oxo-, (S)-*	Nc1ccccc1C(=O)C[C@H](N)C(=O)O
NIST17	*D-Aspartic acid*	N[C@H](CC(=O)O)C(=O)O
NIST17	*.gamma.-L-glutamyl-L-glutamic acid*	N[C@@H](CCC(=O)N[C@@H](CCC(=O)O)C(=O)O)C(=O)O
NIST17	*.gamma.-Benzyl L-glutamate*	N[C@@H](CCC(=O)OCc1ccccc1)C(=O)O
NIST17	*5-Hydroxynorvaline*	NC(CCCO)C(=O)O
NIST17	*3-[3(E)-Hydroxyiminomethyl-4-hydroxyphenyl]-l-alanine*	N[C@@H](Cc1ccc(O)c(/C=N/O)c1)C(=O)O
NIST17	*dl-Cysteine*	NC(CS)C(=O)O

NIST17	*DL-Tyrosine, O-(4-hydroxy-3,5-diiodophenyl)-3,5-diiodo-*	NC(Cc1cc(I)c(Oc2cc(I)c(O)c(I)c2)c(I)c1)C(=O)O
NIST17	*.alpha.-Amino-2,5-dihydro-5-methyl-2-furanaceticacid*	CC1C=CC(C(N)C(=O)O)O1
NIST17	*2-Amino-3-methyl-4-pentynoic acid*	C#CC(C)C(N)C(=O)O
NIST17	*Benzenepropanoic acid, .alpha.-amino-4-hydroxy-2-methyl-*	Cc1cc(O)ccc1CC(N)C(=O)O
NIST17	*dl-2-Phenyltryptophane*	NC(Cc1c(-c2ccccc2)[nH]c2ccccc12)C(=O)O
NIST17	*Valine, 3-[sulfothio]-*	CC(C)(SS(=O)(=O)O)C(N)C(=O)O
NIST17	*Hitidylhistidine*	NC(Cc1cn(C(=O)C(N)Cc2c[nH]cn2)cn1)C(=O)O
NIST17	*6-Amino-l-tryptophan*	Nc1ccc2c(C[C@H](N)C(=O)O)c[nH]c2c1
NIST17	*5-Iodohistidine*	NC(Cc1nc[nH]c1I)C(=O)O
NIST17	*3-Propylnorleucine*	CCCC(CCC)C(N)C(=O)O
NIST17	*dl-3-Phenyl-dl-glutamic acid*	NC(C(=O)O)C(CC(=O)O)c1ccccc1
NIST17	*2-Amino-3-(3-chlorophenyl)propionic acid*	NC(Cc1cccc(Cl)c1)C(=O)O
NIST17	*Tyrosine, 3-fluoro-*	NC(Cc1ccc(O)c(F)c1)C(=O)O
NIST17	*N(6)-(5-(1,2-Dithiolan-3-yl)valeryl)-l-lysine*	N[C@@H](CCCCNC(=O)CCCCC1CCSS1)C(=O)O
NIST17	*L-Cysteine, S-propyl-*	CCCSC[C@H](N)C(=O)O
NIST17	*3,5-Dichlorophenylalanine*	NC(Cc1cc(Cl)cc(Cl)c1)C(=O)O
NIST17	*4-Chlorolysine*	NCCC(Cl)CC(N)C(=O)O
NIST17	*dl-3-Bromophenylalanine*	NC(Cc1cccc(Br)c1)C(=O)O
NIST17	*2-Thiopheneacetic acid, .alpha.-amino-, (.+/-.)-*	NC(C(=O)O)c1cccs1
NIST17	*L-Theanine*	CCNC(=O)CC[C@H](N)C(=O)O
NIST17	*3-Adamantan-1-yl-2-amino-propionic acid*	NC(CC12CC3CC(CC(C3)C1)C2)C(=O)O
NIST17	*l-Buthionine sulfoximine*	CCCCS(=N)(=O)CCC(N)C(=O)O
NIST17	*Benzenebutanoic acid, .alpha.,2-diamino-.gamma.-oxo-*	Nc1ccccc1C(=O)CC(N)C(=O)O
NIST17	*dl-6-Benzyloxytryptophan*	NC(Cc1c[nH]c2cc(OCc3ccccc3)ccc12)C(=O)O
NIST17	*4-Nitro-dl-phenylalanine*	NC(Cc1ccc([N+](=O)[O-])cc1)C(=O)O
NIST17	*D-(-)-Norvaline*	CCC[C@@H](N)C(=O)O
NIST17	*Butanoic acid, 2-amino-4,4-dichloro-*	NC(CC(Cl)Cl)C(=O)O
NIST17	*L-Histidine, 1-methyl-*	Cn1cnc(C[C@H](N)C(=O)O)c1

NIST17	*l-Aspartic acid, .beta.-methyl ester*	COC(=O)C[C@H](N)C(=O)O
NIST17	*S-(2-Benzothiazolyl)cysteine*	NC(CSc1nc2ccccc2s1)C(=O)O
NIST17	*Cyclopropanepropionic acid, .alpha.-amino-.beta.-methyl-2-methylene-*	C=C1CC1C(C)C(N)C(=O)O
NIST17	*5-Fluorodopa*	NC(Cc1cc(O)c(O)c(F)c1)C(=O)O
NIST17	*Histidine, 4-nitro-*	NC(Cc1[nH]cnc1[N+](=O)[O-])C(=O)O
NIST17	*Phenylpropionic acid, .alpha.-amino-2-fluoro-4-hydroxy-5-methoxy-*	COc1cc(CC(N)C(=O)O)c(F)cc1O
NIST17	*2-Amino-2-[3-methyl-2-tetrahydrofuryl]acetic acid*	CC1CCOC1C(N)C(=O)O
NIST17	*6-Heptynoic acid, 2-amino-4-hydroxy-*	C#CCC(O)CC(N)C(=O)O
NIST17	*Propanoic acid, 2-amino-3-(4-methoxybenzylthio)-*	COc1ccc(CSCC(N)C(=O)O)cc1
NIST17	*l-Felinine*	CC(C)(CCO)SCC(N)C(=O)O
NIST17	*dl-4-Phenyl-dl-glutamic acid*	NC(CC(C(=O)O)c1ccccc1)C(=O)O
NIST17	*.alpha.-[5-Methyl-2,3,4,5-tetrahydro-2-furyl]glucine*	CC1CCC(C(N)C(=O)O)O1
NIST17	*.gamma.-dl-Methyl-d-glutamyl-n-butylamine*	CCCCNC(=O)C(C)C[C@@H](N)C(=O)O
NIST17	*l-Tryptophan, 4-(3-methylbutyl)-*	CC(C)CCc1cccc2[nH]cc(C[C@H](N)C(=O)O)c12
NIST17	*4-Nitro-l-phenylalanine*	N[C@@H](Cc1ccc([N+](=O)[O-])cc1)C(=O)O
NIST17	*4-Hexenoic acid, 2-amino-6-hydroxy-4-methyl-*	C/C(=C\CO)CC(N)C(=O)O
NIST17	*5-Hexynoic acid, 2-amino-4-methyl-*	C#CC(C)CC(N)C(=O)O
NIST17	*S-Propylthio-L-cysteine*	CCCSSC[C@H](N)C(=O)O
NIST17	*Phenylpropionic acid, .alpha.-amino-3,4-dimethoxy-.beta.-methyl-*	COc1ccc(C(C)C(N)C(=O)O)cc1OC
NIST17	*S-[2-Aminoethyl]-dl-cysteine*	NCCSCC(N)C(=O)O
NIST17	*Histidine, 4-chloro-2-trifluoromethyl-*	NC(Cc1[nH]c(C(F)(F)F)nc1Cl)C(=O)O
NIST17	*2-Trifluoromethyl-tryptophan*	NC(Cc1c(C(F)(F)F)[nH]c2ccccc12)C(=O)O
NIST17	*2-Aminobutanoic acid, 4-benzylthio-*	NC(CCSCc1ccccc1)C(=O)O
NIST17	*Trihydroxyphenylalanine*	NC(Cc1c(O)cc(O)cc1O)C(=O)O

NIST17	*4-Pyridinepropanoic acid, .alpha.-amino-.beta.-hydroxy-, [R-(R*,S*)]-*	NC(C(=O)O)C(O)c1ccncc1
NIST17	*2-Bromohistidine*	NC(Cc1cnc(Br)[nH]1)C(=O)O
NIST17	*dl-2-.beta.-Thienyl-.alpha.-alanine*	NC(Cc1cccs1)C(=O)O
NIST17	*N-.epsilon.-Acetyl-L-lysine*	CC(=O)NCCCC[C@H](N)C(=O)O
NIST17	*dl-2,6-Diamino-4-hexynoic acid*	NCC#CCC(N)C(=O)O
NIST17	*S-[2-Aminophenyl]-dl-cysteine*	Nc1ccccc1SCC(N)C(=O)O
NIST17	*3-[3(E)-Hydroxyiminomethyl-4-methoxyphenyl]-l-alanine*	COc1ccc(C(/C=N/O)[C@H](N)C(=O)O)cc1
NIST17	*3-Methyl-l-valine*	CC(C)(C)[C@H](N)C(=O)O
NIST17	*Phenylserine, 3-fluoro-4,5-dimethoxy-*	COc1cc(C(O)C(N)C(=O)O)cc(F)c1OC
NIST17	*dl-Erythro-O-methylthreonine*	COC(C)C(N)C(=O)O
NIST17	*Histidine, 2-carboxy-*	NC(Cc1cnc(C(=O)O)[nH]1)C(=O)O
NIST17	*L-Cysteine, S-(triphenylmethyl)-*	N[C@@H](CSC(c1ccccc1)(c1ccccc1)c1ccccc1)C(=O)O
NIST17	*Di-carobenzyloxy-l-4-hydroxylysine*	NC(CC(CCNC(=O)OCc1ccccc1)OC(=O)OCc1ccccc1)C(=O)O
NIST17	*3-(3-Carboxy-4-hydroxyphenyl)-D-alanine*	N[C@H](Cc1ccc(O)c(C(=O)O)c1)C(=O)O
NIST17	*.epsilon.-N-Formyl-L-lysine*	N[C@@H](CCCCNC=O)C(=O)O
NIST17	*2-Amino-3-naphthalen-2-ylpropionic acid*	NC(Cc1ccc2ccccc2c1)C(=O)O
NIST17	*Phenylalanine, 4-acetylamino-3-iodo-*	CC(=O)Nc1ccc(CC(N)C(=O)O)cc1I
NIST17	*D-Lysine*	NCCCC[C@@H](N)C(=O)O
NIST17	*D-2-(4-Hydroxyphenyl)glycine*	NC(C(=O)O)c1ccc(O)cc1
NIST17	*L-Histidine, 3-methyl-*	Cn1cncc1C[C@H](N)C(=O)O
NIST17	*2-Chlorohistidine*	NC(Cc1c[nH]c(Cl)n1)C(=O)O
NIST17	*3-Methoxytyrosine*	COc1cc(CC(N)C(=O)O)ccc1O
NIST17	*Phenylserine, 3,4-dibenzyloxy-*	NC(C(=O)O)C(O)c1ccc(OCc2ccccc2)c(OCc2ccccc2)c1
NIST17	*p-Amino-L-phenylalanine*	Nc1ccc(C[C@H](N)C(=O)O)cc1
NIST17	*.alpha.-[5-Ethyl-2-furyl]glycine*	CCc1ccc(C(N)C(=O)O)o1
NIST17	*[5-Methyl-2-thienyl]glycine*	Cc1ccc(C(N)C(=O)O)s1
NIST17	*(R)-(-)-2-Phenylglycine*	N[C@@H](C(=O)O)c1ccccc1

NIST17	*4-Methyl-dl-tryptophan*	Cc1cccc2[nH]cc(CC(N)C(=O)O)c12
NIST17	*DL-2-Fluorophenylglycine*	NC(C(=O)O)c1ccccc1F
NIST17	*L-Homocitrulline*	NC(=O)NCCCCC(N)C(=O)O
NIST17	*DL-Lysine, 5-hydroxy-*	NCC(O)CCC(N)C(=O)O
NIST17	*DL-Histidine*	NC(Cc1cnc[nH]1)C(=O)O
NIST17	*.gamma.-L-Glutamyl-.alpha.-naphthylamide*	N[C@@H](CCC(=O)Nc1cccc2ccccc12)C(=O)O
NIST17	*Tryptophan, 1-(1,1-dimethylallyl)-, L-*	C=CC(C)(C)n1cc(CC(N)C(=O)O)c2ccccc21
NIST17	*4-Amino-dl-phenylalanine*	Nc1ccc(CC(N)C(=O)O)cc1
NIST17	*N(6)-Carbobenzyloxy-l-4-hydroxylysine*	NC(CC(O)CCNC(=O)OCc1ccccc1)C(=O)O
NIST17	*2-Amino-4-methyl-4-pentenoic acid*	C=C(C)CC(N)C(=O)O
NIST17	*.alpha.-[5-Ethyl-2,3,4,5-tetrahydro-2-furyl]glycine*	CCC1CCC(C(N)C(=O)O)O1
NIST17	*2-Iodohiistidine*	NC(Cc1c[nH]c(I)n1)C(=O)O
NIST17	*S-Benzylcysteine sulfoxide*	NC(CS(=O)Cc1ccccc1)C(=O)O
NIST17	*Butanoic acid, 2,4-diamino-*	NCCC(N)C(=O)O
NIST17	*dl-2-(3-Thienyl)glycine*	NC(C(=O)O)c1ccsc1
NIST17	*s-tert-Butylmercapto-L-cysteine*	CC(C)(C)SSC[C@H](N)C(=O)O
NIST17	*dl-3-Methyl-dl-glutamic acid*	CC(CC(=O)O)C(N)C(=O)O
NIST17	*N(6)-carbobenzyloxy-4,5-dedihydrolysine*	NC(C/C=C/CNC(=O)OCc1ccccc1)C(=O)O
NIST17	*N-(.gamma.-L-Glutamyl)phenylalanine*	N[C@@H](CCC(=O)N[C@@H](Cc1ccccc1)C(=O)O)C(=O)O
NIST17	*l-Cysteine, S-butyl-*	CCCCSC[C@H](N)C(=O)O
NIST17	*3,5-Dibromo-L-tyrosine*	N[C@@H](Cc1cc(Br)c(O)c(Br)c1)C(=O)O
NIST17	*4-Hydroxylysine*	NCCC(O)CC(N)C(=O)O
NIST17	*Histidine, 2-trifluoromethyl-*	NC(Cc1cnc(C(F)(F)F)[nH]1)C(=O)O
NIST17	*Hypoglycin*	C=C1CC1CC(N)C(=O)O
NIST17	*L-Arginine*	N=C(N)NCCCC(N)C(=O)O
NIST17	*S-Carboxymethyl-L-cysteine*	NC(CSCC(=O)O)C(=O)O
NIST17	*Cystine, methyl ester*	COC(=O)C(N)CSSCC(N)C(=O)O
NIST17	*2-Amino-2-[3-methyl-2-furyl]acetic acid*	Cc1ccoc1C(N)C(=O)O
NIST17	*L-Cysteine, S-(diphenylmethyl)-*	N[C@@H](CSC(c1ccccc1)c1ccccc1)C(=O)O

NIST17	*2-(3-Amino-3-carboxy)propyl-4-amino-6-dimethylamino-s-triazine*	CN(C)c1nc(N)nc(CCC(N)C(=O)O)n1
NIST17	*Benzylidene-l-ornithine*	N[C@@H](CCC/N=C/c1ccccc1)C(=O)O
NIST17	*dl-O-Tyrosine*	NC(Cc1ccccc1O)C(=O)O
NIST17	*Butanoic acid, 2-amino-, (S)-*	CCC(N)C(=O)O
NIST17	*dl-2-Aminopimelic acid*	NC(CCCCC(=O)O)C(=O)O
NIST17	*4-Bromohistidine*	NC(Cc1nc[nH]c1Br)C(=O)O
NIST17	*D-Asparagine*	NC(=O)C[C@@H](N)C(=O)O
NIST17	*DL-Glutamine*	NC(=O)CCC(N)C(=O)O
NIST17	*(+)-2-Amino-4-(phenylacetamido)butyric acid*	NC(CCNC(=O)Cc1ccccc1)C(=O)O
NIST17	*L-Asparagine, N.gamma.-[2-(acetylamino)-4-O-[2-(acetylamino)-2-deoxy-3,4,6-tris-O-(trimethylsilyl)-.beta.-D-glucopyranosyl]-2-deoxy-3,6-bis-O-(trimethylsilyl)-.beta.-D-glucopyranosyl]-*	CC(=O)NC1C(NC(=O)C[C@H](N)C(=O)O)OC(CO[Si](C)(C)C)C(O[C@@H]2O[C@@H](CO[Si](C)(C)C)[C@H](O[Si](C)(C)C)[C@@H](O[Si](C)(C)C)[C@@H]2NC(C)=O)C1O[Si](C)(C)C
NIST17	*4-Chlorohistidine*	NC(Cc1[nH]cnc1Cl)C(=O)O
NIST17	*.alpha.-[5-Methyl-2-furyl]glycine*	Cc1ccc(C(N)C(=O)O)o1
NIST17	*.gamma.-L-Glutamyl-4-nitro anilide*	N[C@@H](CCC(=O)Nc1ccc([N+](=O)[O-])cc1)C(=O)O
NIST17	*N-.epsilon.-t-boc-L-Lysine*	CC(C)(C)OC(=O)NCCCC[C@H](N)C(=O)O
NIST17	*Trifluoro-l-methionine*	N[C@@H](CCSC(F)(F)F)C(=O)O
NIST17	*1H-Purine-8-propanoic acid, .alpha.-amino-2,3,6,7-tetrahydro-1,3,7-trimethyl-2,6-dioxo-*	Cn1c(=O)c2c(nc(CC(N)C(=O)O)n2C)n(C)c1=O
NIST17	*Threo-.beta.-methoxy-dl-phenylalanine*	COC(c1ccccc1)C(N)C(=O)O
NIST17	*Threo-p-nitro-.beta.-methoxy-dl-phenylalanine*	COC(c1ccc([N+](=O)[O-])cc1)C(N)C(=O)O
NIST17	*3-(3-Cyano-4-methoxyphenyl)-l-alanine*	COc1ccc(C[C@H](N)C(=O)O)cc1C#N
NIST17	*3,5-Diiodo-L-tyrosine*	N[C@@H](Cc1cc(I)c(O)c(I)c1)C(=O)O

NIST17	*2,4-Dibromohistidine*	NC(Cc1[nH]c(Br)nc1Br)C(=O)O
NIST17	*dl-Homocysteine*	NC(CCS)C(=O)O
NIST17	*4-Hydroxytryptophan*	NC(Cc1c[nH]c2cccc(O)c12)C(=O)O
NIST17	*Adamantan-1-ylaminoacetic acid*	NC(C(=O)O)C12CC3CC(CC(C3)C1)C2
NIST17	*3-Butenoic acid, 2-amino-*	C=CC(N)C(=O)O
NIST17	*Phenylalanine, 4-acetylamino-*	CC(=O)Nc1ccc(CC(N)C(=O)O)cc1
NIST17	*4-Methoxy-l-phenylalanine*	COc1ccc(C[C@H](N)C(=O)O)cc1
NIST17	*2-Amino-3-(2-pyridin-2-yl-ethylsulfanyl)-propionic acid*	NC(CSCCc1ccccn1)C(=O)O
NIST17	*6-Fluorodopa*	NC(Cc1ccc(O)c(O)c1F)C(=O)O
NIST17	*3-Isopropoxy alanine*	CC(C)OCC(N)C(=O)O
NIST17	*l-Homoserine, O-butyl-*	CCCCOCC[C@H](N)C(=O)O
NIST17	*Propanoic acid, 2-amino-3-(4-[4-hydroxy-3-methyl-2-butenyl]-1H-3-indolyl)*	C/C(=C\Cc1cccc2[nH]cc(CC(N)C(=O)O)c12)CO
NIST17	*dl-4-Methyl-dl-glutamic acid*	CC(CC(N)C(=O)O)C(=O)O
NIST17	*DL-Leucine*	CC(C)CC(N)C(=O)O
NIST17	*2-Amino-3-(6-amino-purin-9-yl)-propionic acid*	Nc1ncnc2c1ncn2CC(N)C(=O)O
NIST17	*L-Aspartic acid-.beta.-benzyl ester*	NC(CC(=O)OCc1ccccc1)C(=O)O
NIST17	*L-Cysteine sulfinic acid*	N[C@H](CS(=O)O)C(=O)O
NIST17	*Alanine, 3-amino-*	NCC(N)C(=O)O
NIST17	*L-Glutamic acid 5-methyl ester*	COC(=O)CC[C@H](N)C(=O)O
NIST17	*dl-2-Bromophenylalanine*	NC(Cc1ccccc1Br)C(=O)O
NIST17	*dl-7-Azatryptophan*	NC(Cc1c[nH]c2ncccc12)C(=O)O
NIST17	*6-Nitro-l-tryptophan*	N[C@@H](Cc1c[nH]c2cc([N+](=O)[O-])ccc12)C(=O)O
NIST17	*Carbocysteine, 3TBDMS derivative*	CC(C)(C)[Si](C)(C)NC(CSCC(=O)O[Si](C)(C)C(C)(C)C)C(=O)O[Si](C)(C)C(C)(C)C
NIST17	*D,L-Norleucine, 2TBDMS derivative*	CCCCC(N[Si](C)(C)C(C)(C)C)C(=O)O[Si](C)(C)C(C)(C)C
NIST17	*D-.alpha.-Cyclohexylglycine, N-(tert-butyldimethylsilyl)-, tert-butyldimethylsilyl ester*	CC(C)(C)[Si](C)(C)NC(C(=O)O[Si](C)(C)C(C)(C)C)C1CCCCC1

NIST17	*Fenclonine, 2TBDMS derivative*	CC(C)(C)[Si](C)(C)NC(Cc1ccc(Cl)cc1)C(=O)O[Si](C)(C)C(C)(C)C
NIST17	*Selenomethionine, N,O-bis(tert-butyldimethylsilyl)-*	C[Se]CCC(N[Si](C)(C)C(C)(C)C)C(=O)O[Si](C)(C)C(C)(C)C
NIST17	*DL-Ornithine, 3TBDMS derivative*	CC(C)(C)[Si](C)(C)NCCCC(N[Si](C)(C)C(C)(C)C)C(=O)O[Si](C)(C)C(C)(C)C
NIST17	*m-Fluorophenylalanine, 2TBDMS derivative*	CC(C)(C)[Si](C)(C)NC(Cc1cccc(F)c1)C(=O)O[Si](C)(C)C(C)(C)C
NIST17	*L-Tryptophan, 2TBDMS derivative*	CC(C)(C)[Si](C)(C)NC(Cc1c[nH]c2ccccc12)C(=O)O[Si](C)(C)C(C)(C)C
NIST17	*Methionine sulfone, (DL)-, 2TBDMS derivative*	CC(C)(C)[Si](C)(C)NC(CCS(C)(=O)=O)C(=O)O[Si](C)(C)C(C)(C)C
NIST17	*5-Hydroxylysine, 4TBDMS derivative*	CC(C)(C)[Si](C)(C)NCC(CCC(N[Si](C)(C)C(C)(C)C)C(=O)O[Si](C)(C)C(C)(C)C)O[Si](C)(C)C(C)(C)C
NIST17	*Homocysteine, 3TBDMS derivative*	CC(C)(C)[Si](C)(C)NC(CCS[Si](C)(C)C(C)(C)C)C(=O)O[Si](C)(C)C(C)(C)C
NIST17	*1-Methyl-L-histidine, 2TBDMS derivative*	Cn1cnc(CC(N[Si](C)(C)C(C)(C)C)C(=O)O[Si](C)(C)C(C)(C)C)c1
NIST17	*3-Iodo-L-tyrosine, 3TBDMS derivative*	CC(C)(C)[Si](C)(C)NC(Cc1ccc(O[Si](C)(C)C(C)(C)C)c(I)c1)C(=O)O[Si](C)(C)C(C)(C)C
NIST17	*L-Ornithine, 3TBDMS derivative*	CC(C)(C)[Si](C)(C)NCCC[C@H](N[Si](C)(C)C(C)(C)C)C(=O)O[Si](C)(C)C(C)(C)C

NIST17	*3-Hydroxy-L-tyrosine, 4TBDMS derivative*	CC(C)(C)[Si](C)(C)N[C@@H](Cc1ccc(O[Si](C)(C)C(C)(C)C)c(O[Si](C)(C)C(C)(C)C)c1)C(=O)O[Si](C)(C)C(C)(C)C
NIST17	*tert-Butyldimethylsilyl 2-[(tert-butyldimethylsilyl)amino]-3-(4-[(tert-butyldimethylsilyl)oxy]-3-chlorophenyl)propanoate*	CC(C)(C)[Si](C)(C)NC(Cc1ccc(O[Si](C)(C)C(C)(C)C)c(Cl)c1)C(=O)O[Si](C)(C)C(C)(C)C
NIST17	*L-3-Methylhistidine, 2TBDMS derivative*	Cn1cncc1CC(N[Si](C)(C)C(C)(C)C)C(=O)O[Si](C)(C)C(C)(C)C
NIST17	*L-Tryptophan, 3TBDMS derivative*	CC(C)(C)[Si](C)(C)N[C@@H](Cc1cn([Si](C)(C)C(C)(C)C)c2ccccc12)C(=O)O[Si](C)(C)C(C)(C)C
NIST17	*p-Fluorophenylalanine, 2TBDMS derivative*	CC(C)(C)[Si](C)(C)NC(Cc1ccc(F)cc1)C(=O)O[Si](C)(C)C(C)(C)C
NIST17	*Butanoic acid, 2-[(tert-butyldimethylsilyl)amino]-4-[(tert-butyldimethylsilyl)sulfo]-, tert-butyldimethylsilyl ester, (S)-*	CC(C)(C)[Si](C)(C)NC(CCS(=O)(=O)O[Si](C)(C)C(C)(C)C)C(=O)O[Si](C)(C)C(C)(C)C
NIST17	*O-Benzyl-L-serine, 2TBDMS derivative*	CC(C)(C)[Si](C)(C)NC(COCc1ccccc1)C(=O)O[Si](C)(C)C(C)(C)C
NIST17	*Glufosinate, N,O,O-tris(tert-butyldimethylsilyl)deriv.*	CC(C)(C)[Si](C)(C)NC(CCP(C)(=O)O[Si](C)(C)C(C)(C)C)C(=O)O[Si](C)(C)C(C)(C)C
NIST17	*3-Hydroxy-DL-tyrosine, 4TBDMS derivative*	CC(C)(C)[Si](C)(C)NC(Cc1ccc(O[Si](C)(C)C(C)(C)C)c(O[Si](C)(C)C(C)(C)C)c1)C(=O)O[Si](C)(C)C(C)(C)C
NIST17	*DL-Homophenylalanine, (S)-, 2TBDMS derivative*	CC(C)(C)[Si](C)(C)NC(CCc1ccccc1)C(=O)O[Si](C)(C)C(C)(C)C

NIST17	*DL-2-Fluorophenylglycine, 2TBDMS derivative*	CC(C)(C)[Si](C)(C)NC(C(=O)O[Si](C)(C)C(C)(C)C)c1ccccc1F
NIST17	*DL-Homoserine, 3TBDMS derivative*	CC(C)(C)[Si](C)(C)NC(CCO[Si](C)(C)C(C)(C)C)C(=O)O[Si](C)(C)C(C)(C)C
NIST17	*2-Aminoadipic acid, 3TBDMS derivative*	CC(C)(C)[Si](C)(C)NC(CCCC(=O)O[Si](C)(C)C(C)(C)C)C(=O)O[Si](C)(C)C(C)(C)C
NIST17	*DL-Norvaline, 2TBDMS derivative*	CCCC(N[Si](C)(C)C(C)(C)C)C(=O)O[Si](C)(C)C(C)(C)C
NIST17	*O-Methyl-DL-serine, N-(tert-butyldimethylsilyl)-, tert-butyldimethylsilyl ester*	COCC(N[Si](C)(C)C(C)(C)C)C(=O)O[Si](C)(C)C(C)(C)C
NIST17	*Kynurenine, 2TBDMS derivative*	CC(C)(C)[Si](C)(C)NC(CC(=O)c1ccccc1N)C(=O)O[Si](C)(C)C(C)(C)C
MoNA24GCTOF	3-acetyloxy-2-aminopropanoic acid	CC(=O)OCC(N)C(=O)O
MoNA24GCTOF	(2S)-3-acetyloxy-2-aminopropanoic acid	CC(=O)OC[C@H](N)C(=O)O
MoNA24GCTOF	2-amino-3-methylbutanedioic acid	CC(C(=O)O)C(N)C(=O)O
MoNA24GCTOF	(2S)-2-amino-3-methyl-3-sulfanylbutanoic acid	CC(C)(S)[C@@H](N)C(=O)O
MoNA24GCTOF	2-amino-3-methylbutanoic acid	CC(C)C(N)C(=O)O
MoNA24GCTOF	(2R)-2-amino-3-[[(2R)-2,3-bis[(3R,7R,11R)-3,7,11,15-tetramethylhexadecoxy]propoxy]-hydroxyphosphoryl]oxypropanoic acid	CC(C)CCC[C@@H](C)CCC[C@@H](C)CCC[C@@H](C)CCOC[C@H](COP(=O)(O)OC[C@@H](N)C(=O)O)OCC[C@H](C)CCC[C@H](C)CCC[C@H](C)CCCC(C)C
MoNA24GCTOF	(2S)-2-amino-4-methylpentanoic acid	CC(C)C[C@H](N)C(=O)O
MoNA24GCTOF	(2S)-2-amino-3-methylbutanoic acid	CC(C)[C@H](N)C(=O)O
MoNA24GCTOF	2-aminopropanoic acid	CC(N)C(=O)O
MoNA24GCTOF	2-amino-3-hydroxybutanoic acid	CC(O)C(N)C(=O)O
MoNA24GCTOF	2-amino-3-phosphonooxybutanoic acid	CC(OP(=O)(O)O)C(N)C(=O)O

MoNA24GCTOF	2-aminopentanoic acid	CCCC(N)C(=O)O
MoNA24GCTOF	2-aminohexanoic acid	CCCCC(N)C(=O)O
MoNA24GCTOF	(2S)-2-aminohexanoic acid	CCCC[C@H](N)C(=O)O
MoNA24GCTOF	(2S)-2-aminopentanoic acid	CCC[C@H](N)C(=O)O
MoNA24GCTOF	2-amino-4-ethylsulfanylbutanoic acid	CCSCCC(N)C(=O)O
MoNA24GCTOF	(2S,3S)-2-amino-3-methylpentanoic acid	CC[C@H](C)[C@H](N)C(=O)O
MoNA24GCTOF	(2S)-2-aminobutanoic acid	CC[C@H](N)C(=O)O
MoNA24GCTOF	(2S)-2-amino-5-[[amino(dimethylamino)methylidene]amino]pentanoic acid	CN(C)C(=N)NCCC[C@H](N)C(=O)O
MoNA24GCTOF	2-(methylamino)propanoic acid	CNC(C)C(=O)O
MoNA24GCTOF	(2S)-2-amino-3-(methylamino)propanoic acid	CNC[C@H](N)C(=O)O
MoNA24GCTOF	(2S)-2-(methylamino)propanoic acid	CN[C@@H](C)C(=O)O
MoNA24GCTOF	(2S)-2-(methylamino)pentanedioic acid	CN[C@@H](CCC(=O)O)C(=O)O
MoNA24GCTOF	methyl 2-amino-3-(4-hydroxyphenyl)propanoate	COC(=O)C(N)Cc1ccc(O)cc1
MoNA24GCTOF	methyl (2S)-2-amino-3-phenylpropanoate	COC(=O)[C@@H](N)Cc1ccccc1
MoNA24GCTOF	(2S)-2-amino-4-methylsulfonylbutanoic acid	CS(=O)(=O)CC[C@H](N)C(=O)O
MoNA24GCTOF	2-amino-4-methylsulfinylbutanoic acid	CS(=O)CCC(N)C(=O)O
MoNA24GCTOF	(2S)-2-amino-4-methylsulfinylbutanoic acid	CS(=O)CC[C@H](N)C(=O)O
MoNA24GCTOF	2-amino-4-methylsulfanylbutanoic acid	CSCCC(N)C(=O)O
MoNA24GCTOF	(2S)-2-amino-4-methylsulfanylbutanoic acid	CSCC[C@H](N)C(=O)O
MoNA24GCTOF	(2S)-2-amino-3-methylsulfanylpropanoic acid	CSC[C@@H](N)C(=O)O
MoNA24GCTOF	(2R)-2-amino-3-methylsulfanylpropanoic acid	CSC[C@H](N)C(=O)O
MoNA24GCTOF	(2R,3R)-2-amino-3-hydroxybutanoic acid	C[C@@H](O)[C@@H](N)C(=O)O
MoNA24GCTOF	(2S,3R)-2-amino-3-hydroxybutanoic acid	C[C@@H](O)[C@H](N)C(=O)O
MoNA24GCTOF	(2S,3S)-2-amino-3-methylbutanedioic acid	C[C@H](C(=O)O)[C@H](N)C(=O)O

MoNA24GCTOF	(2S)-2-aminopropanoic acid	C[C@H](N)C(=O)O
MoNA24GCTOF	(2S,3S)-2-amino-3-hydroxybutanoic acid	C[C@H](O)[C@H](N)C(=O)O
MoNA24GCTOF	[(5S)-5-amino-5-carboxypentyl]-trimethylazanium	C[N+](C)(C)CCCC[C@H](N)C(=O)O
MoNA24GCTOF	(2S)-2-amino-4-[[(2S,3S,4R,5R)-5-(6-aminopurin-9-yl)-3,4-dihydroxyoxolan-2-yl]methyl-methylsulfonio]butanoate	C[S+](CC[C@H](N)C(=O)[O-])C[C@H]1O[C@@H](n2cnc3c(N)ncnc32)[C@H](O)[C@@H]1O
MoNA24GCTOF	2-amino-3-methylselanylpropanoic acid	C[Se]CC(N)C(=O)O
MoNA24GCTOF	(2R)-2-amino-3-methylselanylpropanoic acid	C[Se]C[C@H](N)C(=O)O
MoNA24GCTOF	2-amino-3-(1H-imidazol-5-yl)propanoic acid;hydrate;hydrochloride	Cl.NC(Cc1cnc[nH]1)C(=O)O.O
MoNA24GCTOF	2-amino-3-(1-methylimidazol-4-yl)propanoic acid	Cn1cnc(CC(N)C(=O)O)c1
MoNA24GCTOF	2-amino-3-cyanopropanoic acid	N#CCC(N)C(=O)O
MoNA24GCTOF	(2S)-2-amino-3-cyanopropanoic acid	N#CC[C@H](N)C(=O)O
MoNA24GCTOF	(2S)-2-amino-5-(diaminomethylideneamino)pentanoic acid	N=C(N)NCCC[C@H](N)C(=O)O
MoNA24GCTOF	2-amino-4-(diaminomethylideneamino)oxybutanoic acid	N=C(N)NOCCC(N)C(=O)O
MoNA24GCTOF	(2S)-2-amino-4-(diaminomethylideneamino)oxybutanoic acid	N=C(N)NOCC[C@H](N)C(=O)O
MoNA24GCTOF	2,4-diamino-4-oxobutanoic acid	N=C(O)CC(N)C(=O)O
MoNA24GCTOF	(2R)-2,5-diamino-5-oxopentanoic acid	N=C(O)CC[C@@H](N)C(=O)O
MoNA24GCTOF	(2S)-2,5-diamino-5-oxopentanoic acid	N=C(O)CC[C@H](N)C(=O)O
MoNA24GCTOF	(2S)-2,4-diamino-4-oxobutanoic acid	N=C(O)C[C@H](N)C(=O)O
MoNA24GCTOF	2-amino-5-(carbamoylamino)pentanoic acid	N=C(O)NCCCC(N)C(=O)O
MoNA24GCTOF	(2S)-2-amino-6-(carbamoylamino)hexanoic acid	N=C(O)NCCCC[C@H](N)C(=O)O
MoNA24GCTOF	(2S)-2-amino-5-(carbamoylamino)pentanoic acid	N=C(O)NCCC[C@H](N)C(=O)O
MoNA24GCTOF	2-amino-3-hydroxybutanedioic acid	NC(C(=O)O)C(O)C(=O)O
MoNA24GCTOF	2-amino-2-(3,5-dihydroxyphenyl)acetic acid	NC(C(=O)O)c1cc(O)cc(O)c1
MoNA24GCTOF	2-amino-2-phenylacetic acid	NC(C(=O)O)c1ccccc1
MoNA24GCTOF	2-aminobutanedioic acid	NC(CC(=O)O)C(=O)O
MoNA24GCTOF	2-aminopentanedioic acid	NC(CCC(=O)O)C(=O)O

MoNA24GCTOF	2-aminohexanedioic acid	NC(CCCC(=O)O)C(=O)O
MoNA24GCTOF	2-amino-4-sulfobutanoic acid	NC(CCS(=O)(=O)O)C(=O)O
MoNA24GCTOF	2-amino-3-hydroxypropanoic acid	NC(CO)C(=O)O
MoNA24GCTOF	2-amino-3-phosphonopropanoic acid	NC(CP(=O)(O)O)C(=O)O
MoNA24GCTOF	2-amino-3-(5-hydroxy-1H-indol-3-yl)propanoic acid	NC(Cc1c[nH]c2ccc(O)cc12)C(=O)O
MoNA24GCTOF	2-amino-3-(1H-indol-3-yl)propanoic acid	NC(Cc1c[nH]c2ccccc12)C(=O)O
MoNA24GCTOF	2-amino-3-(4-hydroxyphenyl)propanoic acid	NC(Cc1ccc(O)cc1)C(=O)O
MoNA24GCTOF	2-amino-3-phenylpropanoic acid	NC(Cc1ccccc1)C(=O)O
MoNA24GCTOF	3-aminooxolan-2-one	NC1CCOC1=O
MoNA24GCTOF	(2S)-2,6-diaminohexanoic acid	NCCCC[C@H](N)C(=O)O
MoNA24GCTOF	(2S)-2,5-diaminopentanoic acid	NCCC[C@H](N)C(=O)O
MoNA24GCTOF	(2S)-2,4-diaminobutanoic acid	NCC[C@H](N)C(=O)O
MoNA24GCTOF	(2R,3R)-2-amino-3-hydroxybutanedioic acid	N[C@@H](C(=O)O)[C@@H](O)C(=O)O
MoNA24GCTOF	(2R,3R,4S,5R)-2-amino-3,4,5,6-tetrahydroxyhexanoic acid	N[C@@H](C(=O)O)[C@@H](O)[C@H](O)[C@H](O)CO
MoNA24GCTOF	(2S)-2-aminobutanedioic acid	N[C@@H](CC(=O)O)C(=O)O
MoNA24GCTOF	(2S)-2-aminopentanedioic acid	N[C@@H](CCC(=O)O)C(=O)O
MoNA24GCTOF	(2S)-2-amino-5-[[(1R)-1-carboxy-2-sulfanylethyl]amino]-5-oxopentanoic acid	N[C@@H](CCC(O)=N[C@@H](CS)C(=O)O)C(=O)O
MoNA24GCTOF	(2S)-2-aminohexanedioic acid	N[C@@H](CCCC(=O)O)C(=O)O
MoNA24GCTOF	(2S)-2-amino-6-[[(2R,3S,4S,5R)-2,3,4-trihydroxy-5-(hydroxymethyl)oxolan-2-yl]methylamino]hexanoic acid	N[C@@H](CCCCNC[C@@]1(O)O[C@H](CO)[C@@H](O)[C@@H]1O)C(=O)O
MoNA24GCTOF	(2S)-2-amino-4-hydroxybutanoic acid	N[C@@H](CCO)C(=O)O
MoNA24GCTOF	(2S)-2-amino-4-(3-carboxypropanoyloxy)butanoic acid	N[C@@H](CCOC(=O)CCC(=O)O)C(=O)O
MoNA24GCTOF	(2S)-2-amino-4-sulfanylbutanoic acid	N[C@@H](CCS)C(=O)O
MoNA24GCTOF	(2S)-2-amino-3-hydroxypropanoic acid	N[C@@H](CO)C(=O)O

MoNA24GCTOF	(2R)-2-amino-3-phosphonopropanoic acid	N[C@@H](CP(=O)(O)O)C(=O)O
MoNA24GCTOF	(2R)-2-amino-3-sulfopropanoic acid	N[C@@H](CS(=O)(=O)O)C(=O)O
MoNA24GCTOF	(2R)-2-amino-3-sulfinopropanoic acid	N[C@@H](CS(=O)O)C(=O)O
MoNA24GCTOF	(2R)-2-amino-3-sulfanylpropanoic acid	N[C@@H](CS)C(=O)O
MoNA24GCTOF	(2S)-2-amino-3-(5-hydroxy-1H-indol-3-yl)propanoic acid	N[C@@H](Cc1c[nH]c2ccc(O)cc12)C(=O)O
MoNA24GCTOF	(2S)-2-amino-3-(1H-indol-3-yl)propanoic acid	N[C@@H](Cc1c[nH]c2ccccc12)C(=O)O
MoNA24GCTOF	(2S)-2-amino-3-(4-hydroxy-3-iodophenyl)propanoic acid	N[C@@H](Cc1ccc(O)c(I)c1)C(=O)O
MoNA24GCTOF	(2S)-2-amino-3-(3,4-dihydroxyphenyl)propanoic acid	N[C@@H](Cc1ccc(O)c(O)c1)C(=O)O
MoNA24GCTOF	(2S)-2-amino-3-(4-hydroxy-3-nitrophenyl)propanoic acid	N[C@@H](Cc1ccc(O)c([N+](=O)[O-])c1)C(=O)O
MoNA24GCTOF	(2S)-2-amino-3-(4-hydroxyphenyl)propanoic acid	N[C@@H](Cc1ccc(O)cc1)C(=O)O
MoNA24GCTOF	(2S)-2-amino-3-(3-hydroxyphenyl)propanoic acid	N[C@@H](Cc1cccc(O)c1)C(=O)O
MoNA24GCTOF	(2S)-2-amino-3-phenylpropanoic acid	N[C@@H](Cc1ccccc1)C(=O)O
MoNA24GCTOF	(2S)-2-amino-3-(1H-imidazol-5-yl)propanoic acid	N[C@@H](Cc1cnc[nH]1)C(=O)O
MoNA24GCTOF	(2S)-2-amino-3-pyrazol-1-ylpropanoic acid	N[C@@H](Cn1cccn1)C(=O)O
MoNA24GCTOF	(2S)-2-amino-2-(4-hydroxyphenyl)acetic acid	N[C@H](C(=O)O)c1ccc(O)cc1
MoNA24GCTOF	(2R)-2-amino-5-oxo-5-phenylmethoxypentanoic acid	N[C@H](CCC(=O)OCc1ccccc1)C(=O)O
MoNA24GCTOF	(2R)-2-amino-4-hydroxybutanoic acid	N[C@H](CCO)C(=O)O
MoNA24GCTOF	(2R)-2-amino-3-(3,4-dihydroxyphenyl)propanoic acid	N[C@H](Cc1ccc(O)c(O)c1)C(=O)O
MoNA24GCTOF	2-amino-4-(2-amino-3-hydroxyphenyl)-4-oxobutanoic acid	Nc1c(O)cccc1C(=O)CC(N)C(=O)O
MoNA24GCTOF	pyrrolidine-2-carboxylic acid	O=C(O)C1CCCN1
MoNA24GCTOF	(2S)-4-hydroxypyrrolidine-2-carboxylic acid	O=C(O)[C@@H]1CC(O)CN1
MoNA24GCTOF	(2S)-pyrrolidine-2-carboxylic acid	O=C(O)[C@@H]1CCCN1
MoNA24GCTOF	(2S,4R)-4-hydroxypyrrolidine-2-carboxylic acid	O=C(O)[C@@H]1C[C@@H](O)CN1

MoNA24GCTOF	(2S)-2-amino-2-deuteriopropanoic acid	[2H][C@@](C)(N)C(=O)O
IOCB24	2-amino-3-(tert-butoxy)butanoic acid	CC(OC(C)(C)C)C(N)C(O)=O
IOCB24	2-amino-5-[(2-methylpropan-2-yl)oxy]-4-[(2-methylpropan-2-yl)oxycarbonyl]-5-oxopentanoic acid	CC(C)(C)OC(=O)C(CC(N)C(O)=O)C(=O)OC(C)(C)C
IOCB24	2-amino-6-oxo-6-(tritylamino)hexanoicacid	NC(CCCC(=O)NC(C1=CC=CC=C1)(C1=CC=CC=C1)C1=CC=CC=C1)C(O)=O
IOCB24	2-amino-3,3-dimethyl-5-[(2-methylpropan-2-yl)oxy]-5-oxopentanoicacid	CC(C)(C)OC(=O)CC(C)(C)C(N)C(O)=O
IOCB24	2-amino-3-methoxypropanoic acid	COCC(N)C(O)=O
IOCB24	5,5-dimethylpyrrolidine-2-carboxylic acid	CC1(C)CCC(N1)C(O)=O
IOCB24	2-amino-5-[bis[(2-methylpropan-2-yl)oxy]phosphoryl]-4-methylpentanoicacid	CC(CC(N)C(O)=O)CP(=O)(OC(C)(C)C)OC(C)(C)C
IOCB24	2-amino-3-ethylsulfanylpropanoic acid	CCSCC(N)C(O)=O
IOCB24	[tert-butyl(dimethyl)silyl]2-[[tert-butyl(dimethyl)silyl]amino]-3-(2,2-dimethylpropoxy)butanoate	CC(OCC(C)(C)C)C(N[Si](C)(C)C(C)(C)C)C(=O)O[Si](C)(C)C(C)(C)C
IOCB24	1-O,1-O-ditert-butyl3-O-[tert-butyl(dimethyl)silyl]3-[[tert-butyl(dimethyl)silyl]amino]propane-1,1,3-tricarboxylate	CC(C)(C)OC(=O)C(CC(N[Si](C)(C)C(C)(C)C)C(=O)O[Si](C)(C)C(C)(C)C)C(=O)OC(C)(C)C
IOCB24	[tert-butyl(dimethyl)silyl]2-[[tert-butyl(dimethyl)silyl]amino]-6-oxo-6-(tritylamino)hexanoate	CC(C)(C)[Si](C)(C)NC(CCCC(=O)NC(C1=CC=CC=C1)(C1=CC=CC=C1)C1=CC=CC=C1)C(=O)O[Si](C)(C)C(C)(C)C
IOCB24	5-O-tert-butyl1-O-[tert-butyl(dimethyl)silyl]2-[[tert-butyl(dimethyl)silyl]amino]-3,3-dimethylpentanedioate	CC(C)(C)OC(=O)CC(C)(C)C(N[Si](C)(C)C(C)(C)C)C(=O)O[Si](C)(C)C(C)(C)C
IOCB24	[tert-butyl(dimethyl)silyl]2-[[tert-butyl(dimethyl)silyl]amino]-3-methoxypropanoate	COCC(N[Si](C)(C)C(C)(C)C)C(=O)O[Si](C)(C)C(C)(C)C
IOCB24	[tert-butyl(dimethyl)silyl]1-[tert-butyl(dimethyl)silyl]-5,5-dimethylpyrrolidine-2-carboxylate	CC(C)(C)[Si](C)(C)OC(=O)C1CCC(C)(C)N1[Si](C)(C)C(C)(C)C

IOCB24	[tert-butyl(dimethyl)silyl]2-[[tert-butyl(dimethyl)silyl]amino]-3-pyrazol-1-ylpropanoate	CC(C)(C)[Si](C)(C)NC(CN1C=CC=N1)C(=O)O[Si](C)(C)C(C)(C)C
IOCB24	[tert-butyl(dimethyl)silyl]4-[bis[(2-methylpropan-2-yl)oxy]phosphoryl]-2-[[tert-butyl(dimethyl)silyl]amino]-3-methylbutanoate	CC(CP(=O)(OC(C)(C)C)OC(C)(C)C)C(N[Si](C)(C)C(C)(C)C)C(=O)O[Si](C)(C)C(C)(C)C
IOCB24	[tert-butyl(dimethyl)silyl]2-[[tert-butyl(dimethyl)silyl]amino]-3-ethylsulfanylpropanoate	CCSCC(N[Si](C)(C)C(C)(C)C)C(=O)O[Si](C)(C)C(C)(C)C
